# *TREM2* mRNA Expression in Leukocytes Is Increased in Alzheimer’s Disease and Schizophrenia

**DOI:** 10.1371/journal.pone.0136835

**Published:** 2015-09-02

**Authors:** Yoko Mori, Yuta Yoshino, Shinichiro Ochi, Kiyohiro Yamazaki, Kentaro Kawabe, Masao Abe, Tomoji Kitano, Yuki Ozaki, Taku Yoshida, Shusuke Numata, Takaaki Mori, Junichi Iga, Norio Kuroda, Tetsuro Ohmori, Shu-ichi Ueno

**Affiliations:** 1 Department of Neuropsychiatry, Molecules and Function, Ehime University Graduate School of Medicine, Toon, Ehime, Japan; 2 Department of Psychiatry, Course of Integrated Brain Sciences, Medical Informatics, Institute of Health Biosciences, The University of Tokushima Graduate School, Kuramoto-cho, Tokushima, Japan; 3 Kuroda Hospital, Masaki-cho, Ehime, Japan; University of Florida, UNITED STATES

## Abstract

*TREM2* and *TYROBP* are causal genes for Nasu–Hakola disease (NHD), a rare autosomal recessive disease characterized by bone lesions and early-onset progressive dementia. *TREM2* forms a receptor signaling complex with *TYROBP*, which triggers the activation of immune responses in macrophages and dendritic cells, and the functional polymorphism of *TREM2* is reported to be associated with neurodegenerative disorders such as Alzheimer’s disease (AD). The objective of this study was to reveal the involvement of *TYROBP* and *TREM2* in the pathophysiology of AD and schizophrenia. *Methods*: We investigated the mRNA expression level of the 2 genes in leukocytes of 26 patients with AD and 24 with schizophrenia in comparison with age-matched controls. Moreover, we performed gene association analysis between these 2 genes and schizophrenia. *Results*: No differences were found in *TYROBP* mRNA expression in patients with AD and schizophrenia; however, *TREM2* mRNA expression was increased in patients with AD and schizophrenia compared with controls (*P* < 0.001). There were no genetic associations of either gene with schizophrenia in Japanese patients. *Conclusion*: *TREM2* expression in leukocytes is elevated not only in AD but also in schizophrenia. Inflammatory processes involving *TREM2* may occur in schizophrenia, as observed in neurocognitive disorders such as AD. *TREM2* expression in leukocytes may be a novel biomarker for neurological and psychiatric disorders.

## Introduction

Nasu–Hakola disease (NHD), also called polycystic lipomembranous osteodysplasia with sclerosing leukoencephalopathy (PLOSL), is an extremely rare autosomal recessive disease characterized by bone lesions and early-onset progressive neurocognitive disorders [1]. NHD is caused by mutations in the triggering receptor expressed on myeloid cell 2 (*TREM2*) on chromosome 6p21.1 or TYRO protein tyrosine kinase binding protein *(TYROBP)* on chromosome 19q13.1 [2]. These genes encode different domains of the same receptor signaling protein in the activation of immune response, called the TREM2/TYROBP signaling cascade. Several studies have shown that TREM2/TYROBP signaling is essential for the development of osteoclasts and dendritic cells under homeostatic conditions [3] and that synaptogenesis is deregulated in TYROBP-deficient mice [4, 5]. However, the absence of immunological derangement in NHD remains enigmatic.

*TREM2* is also known to be associated with Alzheimer’s disease (AD) and other neurodegenerative diseases. A functional single nucleotide polymorphism (SNP) in *TREM2* (rs75932628>T, p.R47H) is associated with AD [6], Parkinson’s disease [7], frontotemporal dementia [8], and amyotrophic lateral sclerosis [9]. Moreover, Lue et al. [10] reported that *TREM2* expression is upregulated in the brain of patients with AD. Although a vast amount of clinical data has strongly implicated the role of inflammatory and degenerative processes in the pathophysiology of schizophrenia, *TREM2* expression in schizophrenia has not yet been examined. In the present study, we report gene expression and association analyses of both *TYROBP* and *TREM2* in patients with AD and schizophrenia.

## Methods

### Subjects

Descriptive data for each group of participants are shown in Table 1. All participants in this study were of Japanese origin and unrelated to each other.

**Table 1:** Demographic data and clinical characteristics of each group.

**A. Expression analysis for Alzheimer’s disease.**			
	**Alzheimer's disease (n = 26)**	**AD-cnt (n = 26)**	***P*-value**
sex (male: female)	8:18	13:13	0.26
age(years mean ± SD)	79.6 ± 4.0	77.5 ± 4.1	0.07
MMSE score (points mean ± SD)	18.0 ± 5.6	29.7 ± 0.6	< 0.001
CDR number of each grade (0: 0.5: 1: 2: 3)	0: 1: 8: 14: 3	26: 0: 0: 0: 0	< 0.001
**B. Expression analysis for schizophrenia.**			
	**schizophrenia (n = 24)**	**Sc-cnt (n = 24)**	***P*-value**
Sex (male: female)	7:17	7:17	1
age(years mean ± SD)	54.8 ± 18.0	54.8 ± 18.0	1
disease duration (years mean ± SD)	24.2 ± 14.7	none	―
**C. Genotyping analysis.**			
	**schizophrenia (n = 796)**	**control (n = 510)**	***P*-value**
sex (male: female)	457: 339	265: 245	0.06
age(years mean ± SD)	53.0 ± 14.4	35.1 ± 14.2	< 0.001

AD-cnt, the controls against Alzheimer’s disease in expression analysis; MMSE, Mini Mental State Examination; CDR, Clinical Dementia Rating. The score of 0–3 shows classification of dementia (0 = none, 0.5 = questionable, 1 = mild, 2 = moderate, 3 = severe); Sc-cnt, the controls against schizophrenia in expression analysis. The p-value was calculated by student T test, Chi-squired test, and Fisher’s exact test.

#### Participants in AD analysis

We recruited 26 patients with AD [8 males and 18 females, mean age ± standard deviation (SD) = 79.6 ± 4.0 years] from Ehime University Hospital and related community hospitals. AD was diagnosed according to criteria established by the National Institute on Aging and the Alzheimer’s Association and classified as probable AD dementia [11] with bilateral hippocampal atrophy using brain CT or brain MRI findings. Subjects were also assessed by the Mini Mental State Examination (MMSE) [12] and Clinical Dementia Rating (CDR) [13]. The AD control group (AD-cnt) included 22 age-matched elderly participants with normal cognitive function (13 males and 13 females, mean age ± SD = 77.5 ± 4.1 years) who agreed to participate in this study. For inclusion, subjects had to be capable of living independently, have MMSE scores over 28, and be free of cognitive impairment or morphological brain abnormality.

#### Participants in schizophrenia analysis

We recruited 24 patients with schizophrenia (7 males and 17 females, mean age ± SD = 54.8 ± 18.0 years, disease duration at blood draw = 24.2 ± 14.7 years) as well as 24 age-matched controls (Sc-cnt; 7 males and 17 females, mean age ± SD = 54.8 ± 18.0 years) from Ehime University Hospital and related community hospitals. In addition, we enrolled 796 patients with schizophrenia (457 males, 339 females, age = 53.0 ± 3.4 years, 34 patients did not indicate age) who visited Ehime or Tokushima University Hospitals for a gene association study. Schizophrenia was diagnosed according to the Diagnostic and Statistical Manual of Mental Disorders IV criteria by at least 2 certified psychiatrists on the basis of clinical interviews and review of medical records. The comparison group included 510 healthy volunteers (265 males, 245 females, mean age ± SD = 35.1 ± 14.2 years) without psychiatric signs, psychiatric family history, or past history of mental disorders.

#### NHD participant

A 42-year-old male NHD patient homozygous for a *TYROBP* mutation (*TYROBP* c.141delG; *manuscript in preparation*) was included as a reference. We obtained his RNA twice, at intervals of 30 months, and confirmed that both samples expressed equivalent levels of *TYROBP*. We also analyzed his father and mother (aged 72 and 68 years, respectively), both of whom were heterozygous for the *TYROBP* c.141delG mutation.

### Ethical issues

All procedures followed were in accordance with the ethical standards of the responsible committee on human experimentation (institutional and national) and with the Helsinki Declaration of 1964 and later revision. This study was approved by the institutional ethics committees of The Ethics Review Committee for Human Genome/Gene Analysis Research in Ehime University Graduate School of Medicine and the University of Tokushima Graduate School as “Genetic Studies on Neuropsychiatric Diseases” (registry number; 25-K4), and written informed consent was confirmed by all participants or their guardians before the acquisition of blood samples.

### Blood sample collection

Whole peripheral blood samples were collected for the extraction of total RNA and genomic DNA, according to the standard protocol, in PaxGene Blood RNA Systems tubes (BD, Tokyo, Japan) and potassium EDTA tubes, respectively. Absorption spectrophotometry, using NanoDrop-1000 (Thermo Fisher scientific, Yokohama, Japan), was used to determine RNA concentrations and purity (260/280 ratio above 1.8 was used). RNA (1 μg per sample) was reverse-transcribed using the High-Capacity cDNA Reverse Transcription Kit (Applied Biosystems, CA, USA), in a total reaction volume of 40 μL. Genomic DNA was isolated from whole blood leukocytes using the QIAamp DNA Blood Mini Kit (Qiagen, Tokyo, Japan), according to the manufacturer’s protocol.

### Expression analyses

For a quantitative estimate of *TYROBP* and *TREM2* mRNA levels, the StepOnePlus Real-Time PCR System (Applied Biosystems) was used. Specific TaqMan probes were employed (Assay ID: *TYROBP*; Hs00182426_m1, *TREM2*; Hs00219132_m1), and the relative expression level of *TYROBP* or *TREM2* mRNA was determined by comparison with the housekeeping gene *GAPDH* [14–16], serving as an internal standard (all Taqman probes from Applied Biosystems). The final volume reaction was 10 μL using the TaqMan Universal Master Mix (Applied Biosystems). Relative mRNA levels were calculated via the 2^−ΔΔCT^ method [17] using StepOne software (Applied Biosystems). We averaged the fold changes from three wells for each sample (triplicate) and used this average value for statistical analyses. The NHD patient was used for calibration in all experiments to correct the observational error.

### SNP analysis

Three SNPs, rs8113524 (NG_009304.1; assay ID: C___2604899_10), rs3817624 (NG_009304.1; assay ID: C__25603557_20), and rs2234256 (NG_011561.1; assay ID: C__15948232_10) (Table 2)were selected using HaploView v4.2 (Cambridge, MA) as tag SNPs, with squared correlation coefficient (r^2^) between 2 SNPs > 0.8 and minor allele frequency (MAF) > 0.01, on the basis of the current International HapMap project database (http://hapmap.ncbi.nlm.nih.gov/index.html.en). These were analyzed for association study with schizophrenia. Additional SNPs, rs429358 (NG_007084.2; assay ID: C___3084793_20) and rs7412 (NG_007084.2; assay ID: C____904973_10) were used to determine apolipoprotein E (*APOE*) isoforms using a real-time SNP genotyping system (TaqMan Assays, Applied Biosystems). Following this, 1× TaqMan PCR Master Mix, 1× TaqMan SNP genotyping assay, 10 ng genomic DNA, and ultrapure water to a final reaction volume of 6 μL were mixed in each well of an optical plate. Allelic discrimination was performed using StepOnePlus and analyzed using its software.

**Table 2:** Characteristics of the selected two tagging SNPs in *TYROBP* and one tagging SNP in *TREM2*.

No.	Gene	SNP(major/minor allele)	Chromosome Location
1	TYROBP	rs8113524 (T/C)	35905035, Chr 19 intron
2	TYROBP	rs3817624 (C/T)	36398899, Chr 19 intron
3	TREM2	rs2234256 (T/C)	41126655, Chr 6 ex4 (L211P)

### Statistics

Statistical analyses were performed using SPSS v22 (IBM, Tokyo, Japan). Expression levels of *TYROBP* and *TREM2* were compared using the Mann–Whitney U-test and Student’s *t*-test. Correlations of gene expression with age, disease duration, and MMSE score were analyzed using the Spearman test. Fisher’s exact test with simulated *P*-values was used to compare *TREM2* genotype distributions between patients with schizophrenia and controls. Linkage disequilibrium (LD), *TYROBP* haplotypes, genotype distributions, minor allele frequencies, and Hardy–Weinberg equilibrium (HWE) were determined using HaploView v4.2. Statistical power was calculated with G*Power 3 (http://www.gpower.hhu.de/), and statistical significance was defined at the 95% confidence level (*P* = 0.05). The ratio of successful genotyping was over 99%, and we extrapolated the missing values for genotype analysis. Bonferroni corrections were applied to maintain an overall type I error rate of 0.05, taking multiple comparisons into account.

## Results

### *TYROBP* and *TREM2* expression in NHD

The NHD patient homozygous for the *TYROBP* c.141delG mutation exhibited the lowest level of *TYROBP* expression among the study participants, while his parents, heterozygous for the mutation, showed no difference from other groups. In contrast, *TREM2* was expressed in the NHD patient and his parents at the same levels as that in control subjects.

### *TYROBP* and *TREM2* expression in AD and schizophrenia

**Fig 1:**
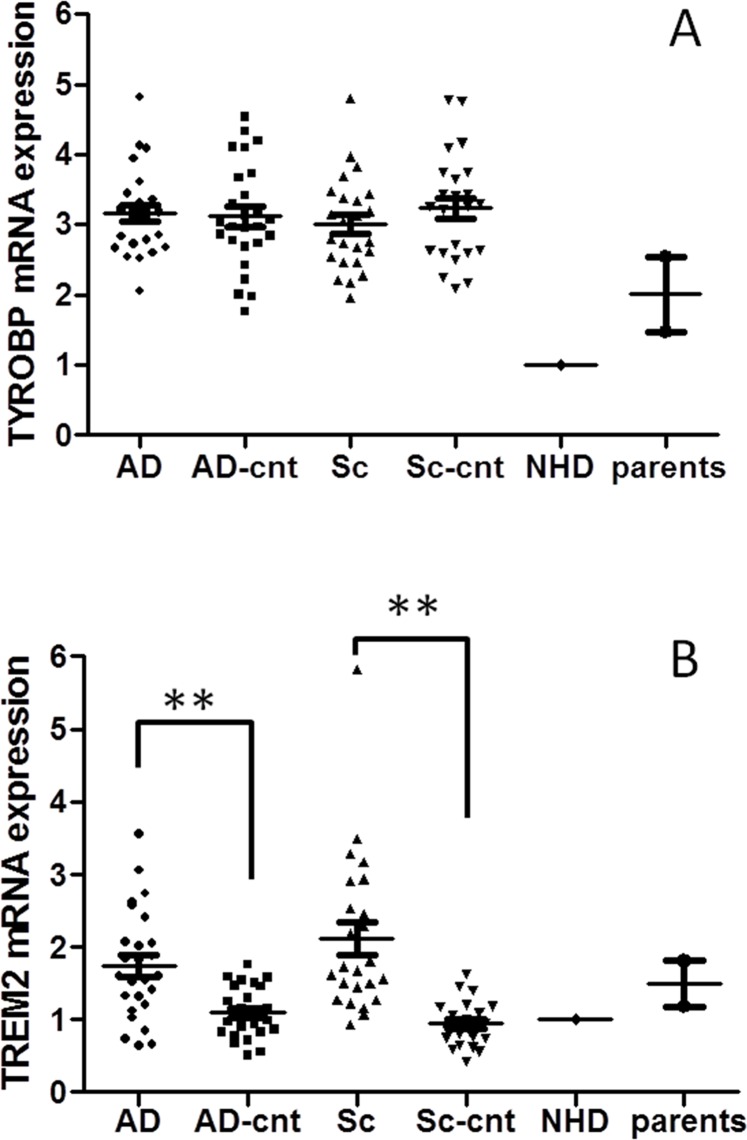
Expression of TYOBP (1A) and TREM2 (1B) in each group. TYROBP expression was the lowest in the NHD patient and showed no difference in other groups.TREM2 expression was higher in schizophrenic and AD patients than in the respective controls. *P* < 0.01 is indicated by ** when the *P* values were calculated by Mann–Whitney U-test.

Fig 1 shows *TYROBP* and *TREM2* expression in leukocytes of patients with AD or schizophrenia and their controls. *TYROBP* expression in patients with AD and schizophrenia was similar to that in their respective control groups (*P* = 0.44 and *P* = 0.13, respectively). In contrast, *TREM2* expression was significantly higher in patients with AD and schizophrenia compared to that in their respective controls (*P* < 0.001 in both cases). This was also confirmed after Bonferroni corrections. There was no correlation between TREM2 expression and other clinical variables such as age (r = 0.08, *P* = 0.71), MMSE (r = 0.01, *P* = 0.95) in AD, and disease duration (r = 0.28, *P* = 0.19) in schizophrenia.

### *TREM2* expression in AD with *APOE* ɛ4

**Fig 2:**
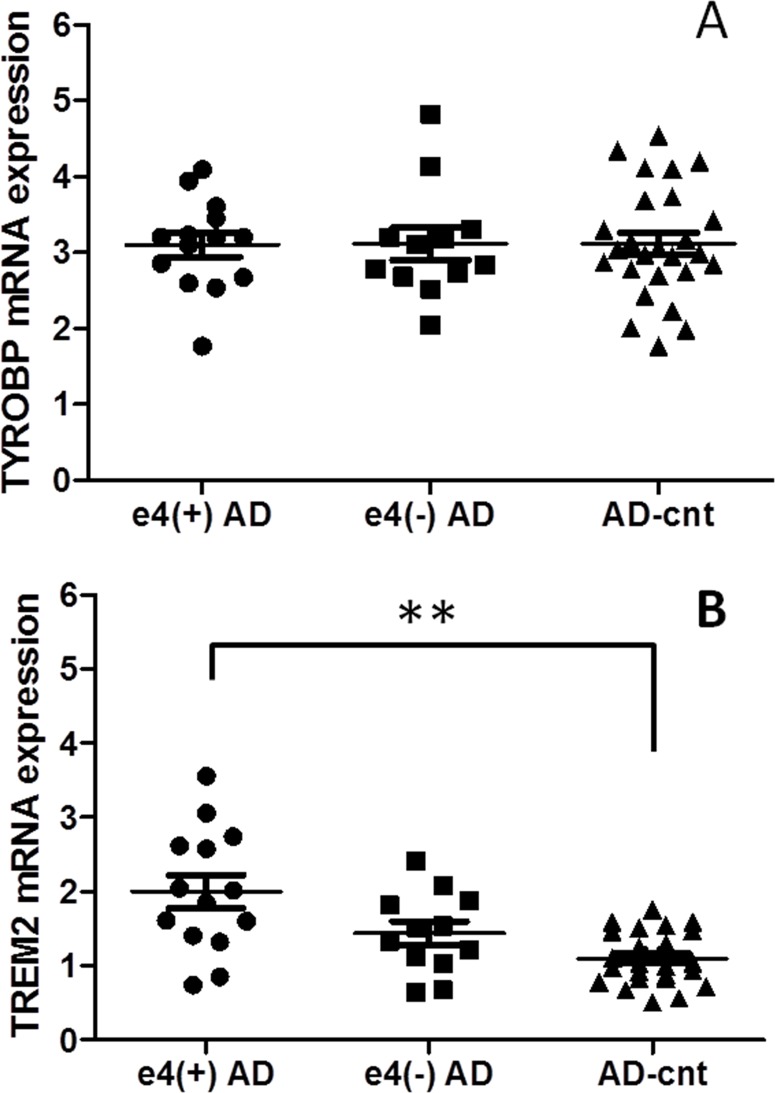
Expression of TYOBP (2A) and TREM2 (2B) in APOE ɛ4-positive AD [ɛ4(+) AD], APOE ɛ4-negative AD [ɛ4(−) AD], and AD-cnt groups. TYROBP expression showed no difference. TREM2 expression was significantly higher in ɛ4-positive AD group than in AD-cnt (including 2 ɛ4-positive subjects) group. The expression levels tended to decline in the following order: ɛ4(+) AD, ɛ4(−) AD, and AD-cnt. *P* < 0.001 after Bonferroni correction is indicated by * * when the *P* values were calculated by Mann–Whitney U-test.

AD patients were typed for the *APOE* ɛ4 allele (Table 3). There were no differences in age, sex, MMSE score, or CDR between the 14 participants (all were ɛ3/ ɛ4) in the *APOE* ɛ4-positive AD group [ɛ4(+) AD] and the 12 participants (all were ɛ3/ ɛ3) in the *APOE* ɛ4-negative AD group [ɛ4(−) AD]. In the AD control group (*n* = 26), only 2 subjects were positive for the *APOE* ɛ4 allele. For these 3 groups, *TYROBP* and *TREM2* mRNA expression levels in leukocytes are shown in Fig 2. In ɛ4(+) AD, *TREM2* expression was significantly higher than that in AD-cnt (*P* < 0.001). Although there were no significant differences between ɛ4(+) and ɛ4(−) AD (*P* = 0.07) or between ɛ4(−) AD and AD-cnt (*P* = 0.07), the relative expression of both genes tended to decrease across groups, in the order ɛ4(+) AD, ɛ4(−)AD, and AD-cnt.

**Table 3:** Characteristics of APOE ɛ4 positive and negative group in AD.

	ɛ4-positive (n = 14)	ɛ4-negative (n = 12)	*P*-value
sex (male: female)	5:09	3:09	0.68
age(years mean ± SD)	80.1 ± 4.3	78.9 ± 3.7	0.45
MMSE score (points mean ± SD)	17.7± 7.1	18.2 ± 3.6	0.84
CDR number of each grade (0: 0.5: 1: 2: 3)	0: 0: 5: 7: 2	0: 1: 3: 7: 1	0.64

AD, Alzheimer’s Disease; MMSE, Mini Mental State Examination; CDR, Clinical Dementia Rating. The score of 0–3 shows classification of dementia (0 = none, 0.5 = questionable, 1 = mild, 2 = moderate, 3 = severe). The *P*-value was calculated by Student *t* test, Chi-squired test, and Fisher’s exact test.

### Case–control association studies in schizophrenia

We performed genotyping of 3 tag SNPs (rs8113524 and rs3817624 in *TYROBP* and rs2234256 in *TREM2*) in 796 patients with schizophrenia and 510 controls (unrelated Japanese participants). The results are shown in Table 4. The result of haplotype analysis between *TYROBP* and schizophrenia is shown in Table 5. In schizophrenia, rs8113524 was not in HWE; however, rs3817624 and rs2234256 were in HWE. No associations between either *TYROBP* or *TREM2* and schizophrenia were revealed by single marker or haplotype analyses.

**Table 4:** Genotyping results of the selected two tagging SNPs in *TYROBP* and one tagging SNP in *TREM2*.

No.	SNP		n (dd/dD/DD)	HWE *P*-value	MAF	single marker *P*-value
1	rs8113524	cases	14/129/651	0.03	0.099	0.74
		controls	2/90/407	0.29	0.095	
2	rs3817624	cases	1/59/734	1	0.038	0.86
		controls	1/36/472	1	0.037	
3	rs2234256	cases	1/11/782	0.1	0.008	0.32
		controls	0/5/501	1	0.005	

d, minor allele; HWE, Hardy‐Weinberg equilibrium; MAF, Minor Allele Frequency

The single marker p-value is calculated by Chi-squared test with omission the missing values.

**Table 5:** Result of association study in the TYROBP gene.

Haplotype	Frequencies	Ratio Counts		Frequencies		P-Value
		case	control	case	control	
TC	0.87	1370.0: 218.0	890.7: 135.3	0.863	0.868	0.69
CC	0.1	157.0: 1431.0	97.3: 928.7	0.099	0.095	0.74
TT	0.04	61.0: 1527.0	38.0: 988.0	0.038	0.037	0.86

*P*-value is calculated by Chi-squared test with omission the missing values.

## Discussion

In NHD, the TREM2/TYROBP signaling pathway is damaged by the mutations in either *TYROBP* or *TREM2*, and this has been suggested to cause defects in microglial activity [18]. It is reported that *TYROBP* expression is normal in the brain of NHD individuals with the *TREM2* mutation [2] and that *TREM2* mRNA extracted from leukocytes is severely reduced in NHD individuals with this mutation [19]. Our results, showing decreased levels of *TYROBP* mRNA and normal levels of *TREM2* mRNA in leukocytes of an NHD patient with the *TYROBP* mutation, are consistent with previous reports.

In the present study, we found that *TREM2* was more highly expressed in leukocytes of patients with AD and schizophrenia, which were of different disease and age groups, whereas *TYROBP* expression was not altered in these groups. Our results may be consistent with the previous study that revealed *TREM2* mRNA expression in monocytes was elevated in AD [16]. In the brain, *TREM2* is primarily expressed in microglia; however, absent or expressed at low levels in granulocytes, monocytes, and macrophages [20]. Kiialainen et al. [21] reported that the microglial expression of *Trem2* was high in cultured mouse neuronal cells. The product of *TREM2* is highly expressed in microglia of the AD brain [10], and *Trem2* expression is particularly high in the amyloid plaques of AD model mice [22]. Higher *TREM2* mRNA levels in *APOE* ɛ4-positive individuals may suggests that *TREM2* expression in leukocytes reflects the AD pathology. Although, unexpectedly, we found no differences between the ɛ4(+) AD and ɛ4(−) AD groups or between the ɛ4(−) AD and AD-cnt groups, it is possible that this is a false-negative result and that differences in expression may be confirmed by increasing the number of subjects.

Hu et al. [16] reported that *TREM2* mRNA and protein levels were significantly higher in monocytes of AD and that the expression was inversely associated with the MMSE score. *TREM2* mRNA was significantly increased in leukocytes of AD in the present study, and we found the tendency to inverse correlation between *TREM2* mRNA expression in leukocytes and the MMSE score in APOE ɛ4-negative AD patients (*P* = 0.08, r = −0.53). We suggest that elevated *TREM2* expression in leukocytes can be utilized as a novel biological marker for AD and may associate with severity in APOE ɛ4-negative AD patients.

Surprisingly, *TREM2* mRNA expression was also elevated in patients with schizophrenia. To date, this is the first report showing *TREM2* upregulation in schizophrenia. Although schizophrenia has been thought of as a non-neurodegenerative disorder, the evidence from several recent reports suggests that schizophrenia may actually be neurodegenerative [23]. Compared to brain tissues, *TREM2* is highly expressed in microglia [24] and reduced microglial activity is reported in Trem2-deficient mice [25]. Although we did not examine inflammatory markers such as C-reactive protein and IL-6, it is well known that C-reactive protein [26, 27] and IL-6 [28] are elevated in schizophrenic patients who are free of overt inflammation. Interestingly, increased microglial activity in schizophrenia was also confirmed by both electron microscopy [29] and positron emission tomography [30]. Higher *TREM2* mRNA levels in leukocytes of schizophrenia may be associated with peripheral inflammation or microglial involvement. Because inflammation in glial cells may lead to neuronal changes in schizophrenia [31], *TREM2* expression in brain samples of patients with schizophrenia should be studied in the future.

*TYROBP* expression was not changed in either AD or schizophrenia. TREM2 in extracellular regions acts as a receptor to bind the ligand [32], whereas TYROBP is present as a second transmembrane receptor and transmits signal [33]. The difference in the role of each domain in TYROBP/TREM2 signaling may explain the different expression patterns between TYROBP and TREM2 in leukocytes. Further studies of the TREM2/TYROBP signaling should be performed to elucidate its role in neurological and psychiatric diseases.

We could not find significant association between TYROBP and TREM2 gene polymorphisms and schizophrenia. In addition, the allele rs8113524 was not present in HWE in schizophrenic patients; this may be due to small population size and low allele frequency [34]. Recently, an association study of 2190 Japanese patients with late-onset AD revealed that *TREM2* variants, including R47H and L211P, were not associated with the disease [35]. Therefore, higher level of *TREM2* mRNA in AD and schizophrenia in this study may not be caused by gene polymorphisms but by other factors such as epigenetic modification.

Our study has several limitations that should be considered. All patients with schizophrenia were taking antipsychotics and all patients with AD were taking cholinesterase inhibitors at the time of the blood sampling. Although our NHD patient was also taking antipsychotics and his *TREM2* mRNA levels were similar to those of controls, we could not exclude the possibility that these pharmacological treatments affected the expression of both genes. Influences of food or physical condition (e.g., body mass index, smoking, infection, and inflammation) on gene expression in leukocytes were not examined. Moreover, it remains to be confirmed whether *TREM2* expression in the brain is in good agreement with that in leukocytes. In addition, future studies should assess the correlation between the severity of inflammation or its markers in central spinal fluid (such as neopterin) and expression of the 2 genes.

In conclusion, we report for the first time that *TREM2* expression in leukocytes is elevated not only in AD but also in schizophrenia and may be a clinical biomarker for these diseases. We believe that our study is an important first step in understanding the role of *TREM2* in the pathophysiology of AD and schizophrenia.
